# Role of Sterically Bulky Azobenzenes in the Molecular Assembly of Pyrene Derivatives: Rectangular Sheet-like Structures and Their Emission Characteristics

**DOI:** 10.3390/ijms24054504

**Published:** 2023-02-24

**Authors:** Pyae Thu, Mina Han

**Affiliations:** Department of Chemistry Education, Kongju National University, 56 Gongjudaehak-ro, Gongju 32588, Republic of Korea

**Keywords:** azobenzene, pyrene, molecular assembly, sheet-like aggregates, emission characteristics

## Abstract

The development of pyrene-based fluorescent assembled systems with desirable emission characteristics by reducing conventional concentration quenching and/or aggregation-induced quenching (ACQ) is highly desirable. In this investigation, we designed a new azobenzene-functionalized pyrene derivative (AzPy) in which sterically bulky azobenzene is linked to pyrene. Absorption and fluorescence spectroscopic results before and after molecular assembly indicate that even in a dilute N,N-dimethylformamide (DMF) solution (~10 μM), AzPy molecules experienced significant concentration quenching, whereas the emission intensities of AzPy DMF-H_2_O turbid suspensions containing self-assembled aggregates were slightly enhanced and showed similar values regardless of the concentration. The shape and size of sheet-like structures, from incomplete flakes less than one micrometer in size to well-completed rectangular microstructures, could be adjusted by changing the concentration. Importantly, such sheet-like structures exhibit concentration dependence of their emission wavelength from blue to yellow-orange. Comparison with the precursor (PyOH) demonstrates that the introduction of a sterically twisted azobenzene moiety plays an important role in converting the spatial molecular arrangements from H- to J-type aggregation mode. Thus, AzPy chromophores grow into anisotropic microstructures through inclined J-type aggregation and high crystallinity, which are responsible for their unexpected emission characteristics. Our findings provide useful insight into the rational design of fluorescent assembled systems.

## 1. Introduction

Photoluminescent organic materials provide a versatile platform for diverse applications, such as light-emitting-diodes, biomedical probes, sensors, optical information storage, and the development of artificial neuron models [1,2,3,4,5,6,7,8,9,10,11,12,13,14,15,16,17,18,19,20,21,22,23,24,25,26,27,28]. Extensive studies on fluorescent assembled nano/microstructures that modify their emission characteristics by changes in molecular geometry of small component molecules, spatial molecular arrangements via intermolecular interactions, and the shape and size of self-assembled aggregates have been reported. To construct fluorescent nano/microstructured materials with desirable functions and properties, it is first necessary to understand the molecular structure of small component fluorophores and their molecular stacking ability via noncovalent intermolecular interactions such as π–π stacking, van der Waals, hydrophobic interactions, hydrogen bonding interactions, etc. [29,30,31,32,33,34,35,36,37].

Pyrene consists of four fused benzene rings and is one of the conventional organic fluorophores. In a dilute solution, its strong monomer emission is detected upon excitation with UV light. However, because the planar π-conjugated molecule tends to pack densely via strong π–π stacking interactions, at increased concentrations and in aggregated states, the excited state dimer or “excimer” formation is promoted, and the corresponding red-shifted emission bands appear at longer wavelengths. Simultaneously, the emission intensity is greatly reduced (i.e., concentration quenching, aggregation-caused quenching: ACQ) [38,39,40,41,42,43,44,45,46,47,48,49,50,51,52,53,54]. H-aggregation is known to cause the ACQ phenomenon, which is an obstacle to practical applications of pyrene-based systems as photoluminescent organic materials. To overcome these drawbacks, it would be desirable to synthesize a new type of pyrene derivative considering the following two aspects: (i) the formation of self-assembled structures via inclined J-type aggregation [38,55,56,57,58] and (ii) the introduction of sterically crowded substituents such as alkyl or cyclodextrin groups [59,60,61] to adjust π–π stacking spacing between pyrene units. Azobenzene is a representative photochromic compound. Light irradiation leads to *trans*↔*cis* molecular conformation changes, exhibiting repeated color changes [62,63,64,65,66,67,68,69,70]. Azobenzene and its derivatives are known to be non-fluorescent because energy in the excited state is consumed by very fast *trans*↔*cis* isomerization, which significantly reduces light emission. Exceptionally, fluorescence is sometimes found from the following azobenzene derivatives: donor–acceptor substituted azobenzenes [62,63,64], *ortho*- and *para*-hydroxy azo compounds [62,63,64,71,72,73], *trans*-blocked azobenzenes that do not undergo photoisomerization [74], and aggregation-induced emission enhancement (AIEE) [62,63,75,76,77,78]. It is known that restriction of intramolecular rotations (RIR) and J-type molecular arrangements associated with planarization suppress the non-radiative deactivation pathway, thereby resulting in enhanced emission in the aggregated state. In addition, flat azobenzene is advantageous for H-type aggregation through relatively strong π–π stacking between aromatic units [62,63,64,69]. In contrast, the introduction of sterically bulky substituents in the *ortho* position significantly distorts *trans*-azobenzene, resulting in a longer interlayer stacking distance [26,72].

There have been limited reports on investigating azobenzene-based photoswitches by connecting pyrene and azobenzene [79,80,81,82,83,84,85,86]. Han and coworkers synthesized a fluorescent compound (PyAzEG) having a pyrene fluorophore directly attached to a twisted azobenzene moiety [82]. Spheres formed via self-assembly showed enhanced emissions compared with monomeric solutions. Recently, Yamauchi et al. reported crystallization-induced emission (CIE)-active pyrene-functionalized azobenzene derivatives [86]. Although these derivatives show negligible emission in solution, the significant suppression of *trans*↔*cis* photoisomerization within microcrystals is responsible for the CIE property.

**Scheme 1:**
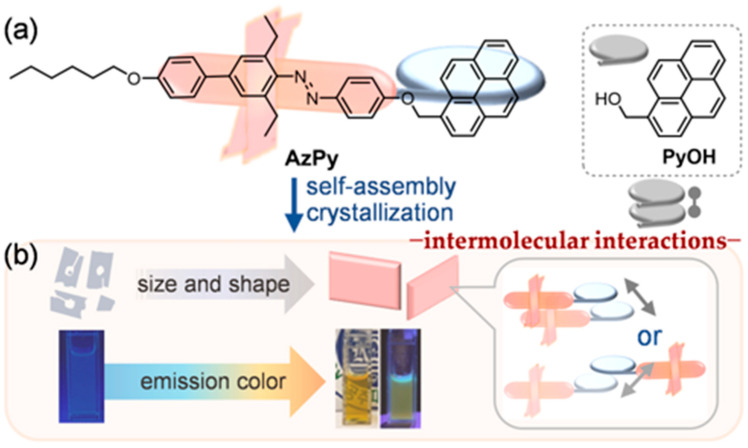
(**a**) Chemical structures of AzPy and PyOH (precursor). (**b**) Schematic representation of the role of sterically bulky (twisted) azobenzene in the self-assembly of AzPy. The introduction of an azobenzene moiety into pyrene plays an important role in the rectangular assembly via inclined π–π stacking interactions and high stacking ability.

To reduce the ACQ effect in the aggregated state and improve the desired emission characteristics, the synthesis strategy of pyrene-based chromophores used in this study involved connecting *ortho*-dialkylated azobenzene to planar pyrene (AzPy, Scheme 1). Comparison with the precursor (PyOH), which is not linked to azobenzene, suggests that the sterically twisted azobenzene moiety plays an important role in converting the spatial molecular arrangements of AzPy molecules from H- to J-type aggregation mode and in helping them to grow into sheet-like microstructures with high crystallinity. The size and completion degree of the birefringent microsheets was readily improved, from incomplete flakes to well-completed rectangular microstructures, by variation of the concentration. Importantly, the molecular assembly into sheet-like aggregates shows concentration-dependent emission color changes from blue to yellow-orange. In addition to their emission characteristics, strong birefringence associated with in-plane molecular arrangements of AzPy molecules was investigated.

## 2. Results and Discussion

### 2.1. Absorption and Emission Spectroscopic Characteristics

**Figure 1:**
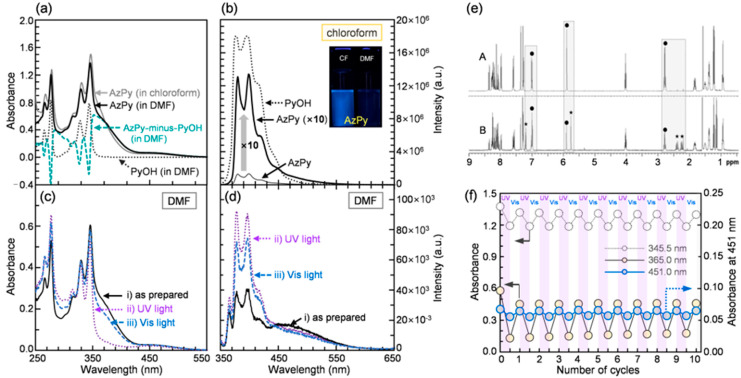
UV-vis absorption and fluorescence spectra of AzPy and PyOH in chloroform and DMF. (**a**) Absorption (20 μM) and (**b**) fluorescence spectra, emission under UV light irradiation (inset). (**c**) Absorption and (**d**) fluorescence (λ_ex_ = 330 nm) spectral changes of AzPy in DMF (10 μM) after UV (at 365 nm, ~1.0 mW/cm^2^) and subsequent visible (at 436 nm, ~1.0 mW/cm^2^) light irradiation. (**e**) ^1^H NMR spectral changes in CDCl_3_. (**A**) As-prepared (all *trans* form). (**B**) After UV light irradiation for 90 min (*trans*/*cis* ≈ 30/70). (**f**) Changes in the absorbances at 345, 365, and 451 nm upon alternating irradiation with UV and visible light.

Figure 1 shows the absorption and fluorescence spectra of AzPy in N,N-dimethylformamide (DMF) and chloroform solutions. Irrespective of the solvent polarity, dilute AzPy solutions (10–20 μM) are light yellow, and five characteristic absorption bands derived from vibrationally structured transitions of the pyrene unit appear in the 260–350 nm wavelength [87,88,89,90]. Compared with the absorption bands (at 266, 277, 314, 328, and 344 nm) of the precursor (PyOH, dotted line in Figure 1a) that is not coupled with the azobenzene moiety, these are red-shifted by 0–1 nm and 1–3 nm in DMF (266, 277, 316, 329 and 345 nm) and chloroform (267, 278, 317, 330 and 347 nm), respectively. Evidently different from PyOH, AzPy has a broad shoulder attributable to the π–π* transition of the azobenzene unit in the 350–400 nm range. Based on the differences in the spectra of AzPy and PyOH (AzPy-minus-PyOH), it is believed that the π–π* band of the azobenzene unit is partially overlapped with the band (at ~345 nm) arising from the S_0_→S_2_ transition of pyrene. The molar extinction coefficient (*ε*) at 365 nm was calculated to be ~25,000 M^−1^ cm^−1^, which is almost identical to those of previously reported *ortho*-alkylated azobenzene derivatives [69]. In addition, a weak featureless band near 450 nm (*ε* ≈ 2800 M^−1^ cm^−1^) corresponds to the partially allowed n–π* transition for the twisted *trans*-azobenzene.

In a relatively nonpolar chloroform solution, AzPy has intense monomer emission maximized at 380 and 398 nm, along with a weak shoulder located at around 417 nm (Figure 1b), but excimer-like emission in the longer wavelength region is hardly detected. This is comparable to the fluorescence spectrum recorded for PyOH. Owing to the suppression of pyrene excited dimer (or excimer) formation, despite the fluorescence quenching ability of azobenzene, AzPy shows violet-blue emission (inset photo in Figure 1b). The fluorescence quantum yield (Φ_f_) measured using pyrene as the reference [36] was found to be ~0.04.

By contrast, even a dilute DMF solution (10 μM) exhibited conspicuous excimer-like emission in the 440–570 nm range, together with significantly weakened monomer emission (Figure 1d). The monomer emission intensity dropped ~15-fold relative to that of the chloroform solution (Figure 1b). This is due to the more favorable intermolecular interactions between hydrophobic pyrene units in the highly polar solvent. As a result, monomer fluorescence quenching caused by strong π–π stacking interactions are detected.

### 2.2. Repeated Trans↔Cis Photoswitching

Repeated light-induced *trans*↔*cis* isomerization was confirmed by UV-visible absorption and ^1^H NMR spectral measurements. Irradiation of the AzPy DMF solution with UV light at 365 nm gave rise to a noticeable reduction in the π–π* absorption band of the azobenzene moiety and, simultaneously, to a ~15–18% decrease of the n–π * absorption band relative to that in the as-prepared *trans* state (Figure 1c and Figure S1a). The amount of *cis*-form present in the photostationary state was then estimated to reach ~70% (Figure 1e). From a comparison with previously reported pyrene-containing azobenzene dyes showing low photoisomerization yields [82], it is surmised that a methoxy linker introduced between azobenzene and pyrene moieties contributed to improving the *trans*-to-*cis* isomerization yield of AzPy. Subsequent irradiation of the UV-exposed AzPy solution with visible light (at 436 nm) restored the π–π* band to ~75% of its initial value; this indicates that the *trans*-AzPy form exists as a major component (*trans*/*cis* ≈ 75/25) at the photostationary state. Moreover, Figure 1f shows repeated variations in the absorbances at 345, 365, and 451 nm. Such excellent *trans*↔*cis* photoswitching was repeatable more than 50 times.

In addition to the absorption spectral changes, irradiation of the 10 μM DMF solution with UV light led to a 2.3-fold increase in monomer emission and a concomitant decrease in excimer emission (Figure 1d). Thus, the *I*_460/378_ value decreased from 0.44 to 0.16. Such fluorescence spectral changes depending on the wavelength of the light stem from a significant number of sterically crowded bent-shaped *cis*-azobenzene interfering with strong π–π stacking interactions between neighboring pyrene moieties, thereby increasing the proportion of monomeric species. Subsequent switching to blue light to promote *cis*-to-*trans* back isomerization reduced the monomer emission intensity again.

### 2.3. Concentration-Dependent Fluorescence Quenching in DMF

**Figure 2:**
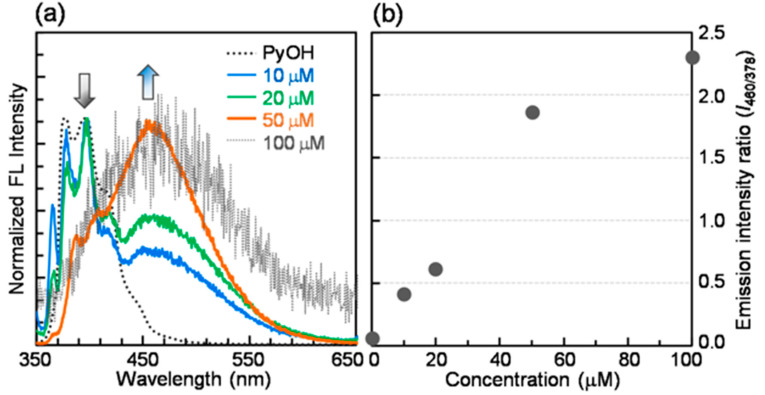
Concentration-dependent fluorescence quenching in DMF solutions. (**a**) Normalized fluorescence spectra (λ_ex_ = 330 nm). (**b**) Emission intensity ratio (*I*_460/378_) of AzPy at different concentrations.

To investigate concentration-dependent fluorescence quenching, we prepared AzPy DMF solutions with concentrations ranging from 10 to 1000 μM. Unlike 10 and 20 μM solutions, which had both monomer and excimer emission bands, a 50 μM solution showed a distinct blue emission band at ~460 nm, together with a negligibly weak shoulder in the 370–420 nm range (Figure 2a). Notably, a more concentrated 100 μM solution was very weakly fluorescent and possessed a single emission band located at ~460 nm, with no monomer emission. Thus, the ratio of the emission intensities at 460 and 378 nm (*I*_460/378_) improved from 0.4 to 2.3 (Figure 2b), indicating a significant increase in pyrene excited dimer formation. Moreover, neither monomer nor excimer emission was detected from solutions concentrated above 200 μM. This suggests that strong intermolecular interactions between planar pyrene units dramatically reduced light emission (i.e., concentration quenching) [91,92].

### 2.4. Self-Assembly into Rectangular Structures

**Figure 3:**
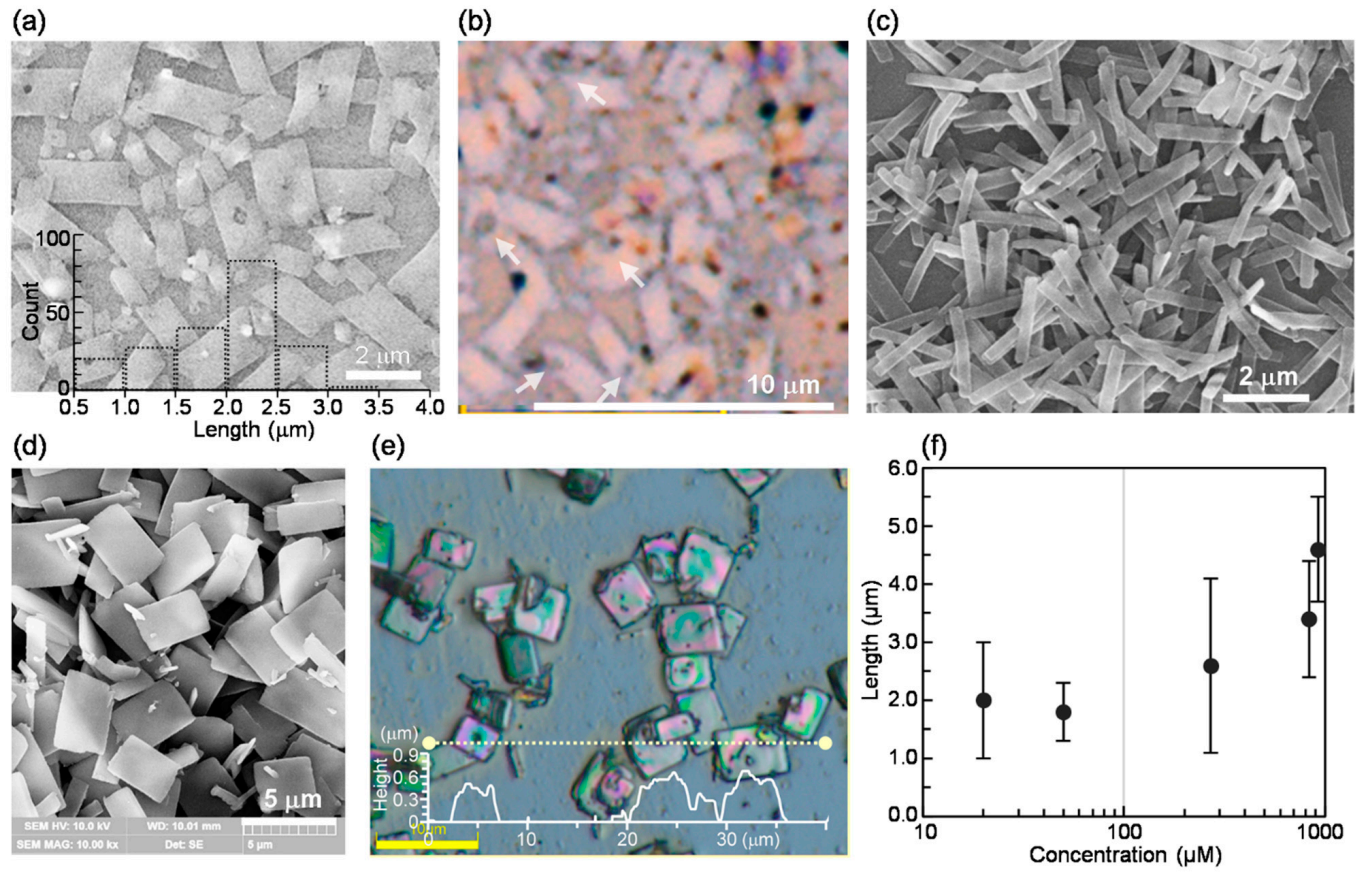
SEM and OM images of rectangular sheet-like structures obtained from (**a**,**b**) 20 μM DMF-H_2_O (1/3, *v*/*v*), (**c**) 50 μM DMF-H_2_O (1/1, *v*/*v*), (**d**,**e**) 920 μM DMF-H_2_O (6/0.5, *v*/*v*) turbid mixtures. (**f**) Changes in the lengths of sheet-like structures as a function of the DMF-H_2_O mixture concentration (counting 200 aggregates). The holes and tears are marked with white arrows in (**b**).

A yellowish suspension was produced when ultrapure water (a poor solvent) was added to an AzPy DMF solution with mild shaking. As confirmed by the scanning electron microscopy (SEM) image in Figure 3a, 20 μM DMF-H_2_O turbid mixtures contained thin flakes with a thickness of less than 1 μm and incomplete sheet-like aggregates with lengths of approximately 1–3 μm. Both sides of the rectangular sheets were frequently torn and uneven. Some sheets had holes. Optical microscopy (OM) observations supported the existence of incomplete aggregates (Figure 3b and Figure S3). Accordingly, micrometer-sized sheets with such defects were not formed due to the vacuum required for SEM sample preparation. Rather, it seems reasonable to interpret that dropping water into a dilute DMF solution produces a large number of crystal nuclei in a very early stage; however, due to the lack of AzPy molecules, molecular assembly presumably stops at such thin flakes without fully developing into rectangular structures.

**Figure 4:**
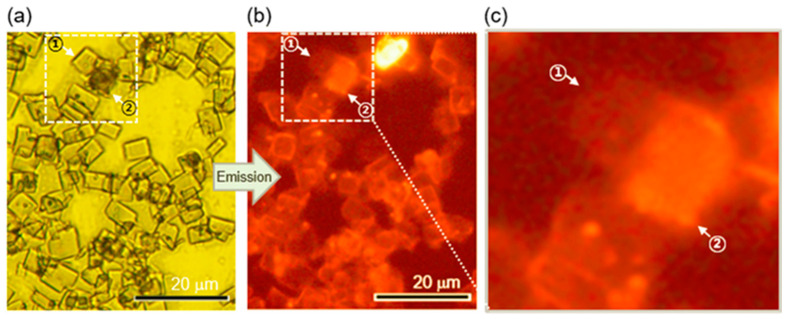
(**a**) OM and corresponding (**b**) FOM images of rectangular microstructures acquired from 920 μM DMF-H_2_O (6/0.5, *v*/*v*) (**c**) The magnified image of the selected area in (**b**).

Indeed, rectangular sheet-like structures with fewer defects were often found in samples produced from concentrated suspensions (Figure S2 and Figure 3c–f). Their length and edge angles were found to be 0.5–3 μm and 85–105 degrees, respectively. The SEM and OM images in Figure 3d–f reveal that the most concentrated suspension (920 μM) contained large microsheets of ~4–6 μm and <0.1 μm size and thickness, respectively. Their edge angles were approximately 82–98°, similar to those of sheets obtained from dilute suspensions. Importantly, such rectangular microsheets showed an orange emission observable with conventional fluorescence optical microscopy (FOMs) (Φ_f_ ~0.003, Figure 4). A comparison of the emission intensities of the microsheets denoted by dotted lines (① and ②) in Figure 4 verifies that a single thin sheet emits very weakly, whereas the area where several sheets overlap shows stronger emission. 

**Figure 5:**
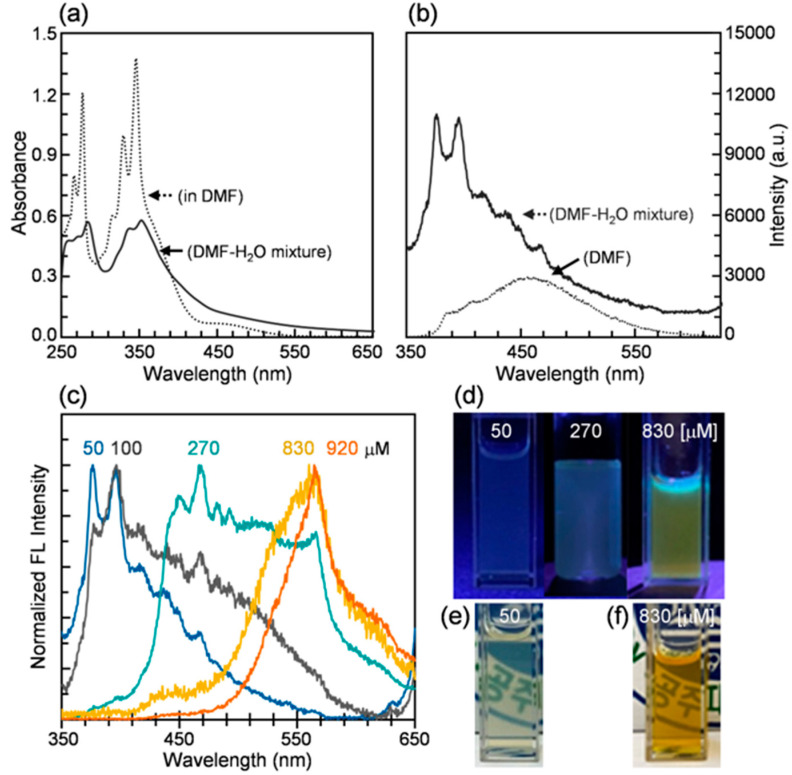
Concentration dependence of emission wavelength: Changes in (**a**) UV-vis absorption spectra before (20 μM in DMF) and after the formation of sheet-like structures (20 μM in DMF-H_2_O). (**b**) Fluorescence spectra in DMF solution (50 μM) and in DMF–H_2_O mixture (50 μM, 1/1, *v*/*v*). (**c**) Normalized fluorescence spectra of turbid suspensions at different concentrations. (**d**) Emission under UV light irradiation. (**e**,**f**) Photographs of turbid DMF-H_2_O mixtures.

Next, to investigate self-assembly into rectangular microstructures and the resulting spectroscopic characteristics, we conducted UV-visible absorption and fluorescence spectroscopy measurements of turbid samples ranging from 20 to 920 μM. As can be clearly seen in Figure 5 (also see Figures S2 and S4), the absorption spectra of the 20, 50, and 100 μM suspensions had high baseline values in the wavelength region of 600 nm or more (indicated as turbidity), indirectly verifying the formation of thin sheet-like aggregates. Relative to those of the transparent DMF solution of the same concentration, five absorption bands appearing in the 260–350 nm range became quite broad and were red-shifted by 2–10 nm. This is in contrast to the PyOH DMF-H_2_O mixtures, which showed a slight blue-shift (Figure S5). These absorption spectral changes before and after the self-assembly appear to support the idea that crystalline sheet-like structures can be created through mutually inclined π–π stacking interactions between planar pyrene moieties (i.e., in a J-type aggregation manner). 

In addition, Figure 5b, Figures S2b and S4b display fluorescence spectral changes measured at different concentrations. For instance, when excited with UV light, a 50 μM milky suspension showed weak monomer emission at around 376 and 396 nm. Together with the abnormally high baselines in the ≥550 nm range, new non-negligible emission bands appeared in the 400–550 nm range. More concentrated 100 and 270 μM DMF-H_2_O suspensions showed blue-green emission and significantly broad emission bands in the wavelength range between 370 and 600 nm (Figure 5c–f). Moreover, the maximum emission wavelengths observed from deep yellow 830 and 920 μM suspensions were further red-shifted to 565 nm and exhibited yellow-orange emission, as shown in the photographs in Figure 5d,f. Evidently, despite the approximately 50-fold increases in AzPy DMF-H_2_O mixture concentration (from 20 to 920 μM), their emission intensities remained almost identical without any noticeable decreasing tendency (Φ_f_ ~ 0.002). 

Thus, based on the absorption and emission spectroscopic results and SEM, OM, and FOM observations, our findings can be summarized as follows. With increasing concentration, (i) the size and completion degree of the crystalline structures improved from incomplete thin flakes to well-completed rectangular microstructures. As a consequence, (ii) with the emergence of new emission bands in the longer wavelength ranges, the emission color changes from blue to yellow-orange, and (iii) stable emission intensities without noticeable fluorescence quenching occurred due to the rectangular assembly.

### 2.5. POM & XRD Measurements

**Figure 6:**
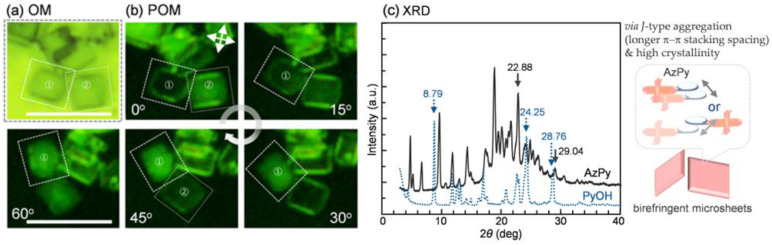
(**a**) OM and corresponding (**b**) POM images of birefringent anisotropic microsheets placed between the crossed polarizers. The sample was rotated by 15 degrees with respect to the crossed polarizers. Scale bar: 10 μm. (**c**) Powder X-ray diffraction (PXRD) patterns of AzPy and PyOH solid samples.

Polarized optical microscopy (POM) used under cross-Nicol conditions is capable of determining birefringent properties of microstructures by monitoring image contrast changes (Figure 6). Figure 6b shows POM images taken by rotating a sample with microsheets by 15 degrees with respect to the polarizer and analyzer. In the thin sheet ① marked by a white dotted line, when one long side is positioned at exactly 0° (i.e., parallel to the polarizer or analyzer), the level of crystal birefringence was found to be maximally dark. Upon being rotated by 15 degrees, the microsheet gradually brightened, reaching maximum brightness at 45°. These contrast changes suggest that AzPy chromophores are regularly arranged along one side of two-dimensional sheets (i.e., in-plane molecular arrangement) [93]. 

Next, to obtain a better understanding of how regularly AzPy molecules are spatially arranged to grow into crystalline microstructures, powder X-ray diffraction (PXRD) measurements were carried out. Figure 6c shows PXRD patterns for the precursor (PyOH) and sheet-like aggregates (AzPy). PyOH has intense and sharp peaks at 2*θ* = 8.79° (d = 10.05 Å), 24.25° (3.67 Å), and 28.76° (3.09 Å). The strongest peak (d = 10.05 Å) corresponds roughly to the molecular length. Notably, the values of 3.67 and 3.09 Å are presumably attributable to interplanar distances associated with strong π–π stacking interactions between planar aromatic units (3.4–3.7 Å) and ArC–H⋯π interactions (2.6–3.1 Å), respectively [44,94,95,96,97,98,99,100,101,102,103]. Combining this XRD pattern and the aforementioned blue-shift of absorption bands for PyOH, disk-like PyOH molecules are likely arranged in more or less an H-type aggregation manner.

By comparison, AzPy crystalline sheet-like microstructures have a strong peak at 2*θ* = 22.88° (d = 3.88 Å) and a negligibly weak signal at 29.04° (3.07 Å). The longer value of 3.88 Å presumably corresponds to an inclined interlayer stacking distance between neighboring pyrene units. The reason for the longer π–π intermolecular interaction spacing is attributed to the introduction of a highly twisted *ortho*-dialkylated azobenzene moiety, which requires a large, occupied area per molecule, which is in good agreement with the values in the literature [104,105,106]. As a consequence, the ArC–H⋯π interactions could occur at a distance in a range of 3.50–3.57 Å (2*θ* ~ 25°), longer than 3.07 Å.

Based on the red-shift of the pyrene-based absorption and emission bands and the relatively longer interplanar spacing, we conclude that the AzPy chromophores grow into rectangular sheet-like aggregates through inclined J-type aggregation and high stacking ability. Importantly, even after rectangular assembly, their emission intensities remained stable without conventional ACQ. The interesting concentration-dependent emission wavelength appears to be closely associated with the size and shape changes of the sheet-like aggregates formed in polar DMF-H_2_O mixed systems.

Additionally, whereas the AzPy chromophores underwent excellent *trans*↔*cis* photoswitching in response to light wavelengths in solutions, no evident light-induced molecular switches were monitored in turbid suspensions or in the solid samples. These results can be supported by the interpretation that AzPy molecules exist in a frozen state through highly regular molecular arrangements over micrometer-sized regions.

## 3. Materials and Methods 

### 3.1. Synthesis of AzPy

AzPy was synthesized by reacting the precursor (AzOH) (0.25 g, 0.58 mmol) with 1-bromomethylpyrene (PyBr, see Supplementary Materials, Figure S9) (0.26 g, 0.87 mmol) in N,N-dimethylformamide (15 mL) in the presence of K_2_CO_3_ (0.24 g, 1.74 mmol) [107,108]. The reaction mixture was stirred at 110 °C for 24 h and cooled down to room temperature. Water and chloroform were added to separate the organic layer. The collected residue from rotatory evaporation was purified by silica gel column chromatography (n-hexane: chloroform, *v*/*v* = 1/3). (0.10 g, yield: 27%).

^1^H NMR (400 MHz, CDCl_3_) δ 8.36 (d, 1H, Py*H*, J = 9.22), 8.25–8.28 (m, 3H, Py*H*), 8.13–8.24 (m, 5H, Py*H),* 8.08 (t, 1H, Ar*H*), 7.98 (d, 2H, Ar*H*, J = 8.98 Hz), 7.59 (d, 2H, Ar*H*, J = 8.80 Hz), 7.36 (s, 2H, Ar*H*), 7.27 (s, 1H, Ar*H*), 7.01 (d, 4H, Ar*H*, J = 8.93 Hz), 5.89 (s, 2H, ArOC*H_2_*Py), 4.04 (t, 2H, ArOC*H_2_*(CH_2_)_4_CH_3_), 2.79 (q, 4H, ArC*H_2_*CH_3_), 1.84 (quin, 2H, ArOCH_2_C*H_2_*(CH_2_)_3_CH_3_), 1.52 (quin, 2H, ArO(CH_2_)_2_C*H_2_*(CH_2_)_2_CH_3_), 1.37–1.41 (m, 4H, ArO(CH_2_)_3_(C*H_2_*)*_2_*CH_3_), 1.24 (t, 6H, ArCH_2_C*H_3_*), 0.950 (t, 3H, ArO(CH_2_)_5_C*H_3_*). ^13^C NMR (400 MHz, CDCl_3_) δ 161.3, 158.8, 149.8, 147.6, 140.4, 137.2, 133.3, 131.8, 131.3, 130.8, 129.4, 129.2, 128.3, 127.8, 127.4, 126.9, 126.2, 125.9, 125.6, 125.5, 124.7, 124.5, 122.9, 115.3, 114.8, 69.2, 68.1, 31.6, 29.3, 25.8, 25.6, 22.6, 15.7, 14.1 (Figures S7 and S8). HRMS (m/z): [M + H] found: 645.3481 (=M + 1), Calcd for C_45_H_45_N_2_O_2_ = 645.3436.

### 3.2. Materials

The reagents and solvents for the synthesis and purification were purchased from Aldrich and SamChun Pure Chemical Co. Ltd., Pyeongtaek-si, Republic of Korea. After dissolving the purified compound in Deuterated CDCl_3_ solvent, ^1^H and ^13^C NMR spectra were recorded with Bruker 400 MHz NMR spectrometer. The spectroscopic grade N,N-dimethylformamide (DMF) was obtained from Kanto Chemical Co., Inc., Tokyo, Japan, and ultrapure water (which was purified to reach a minimum resistivity of 18.0 MΩ∙cm (25 °C) using a µPure HIQ water purification system, Romax, Hanam, Republic of Korea) was used in all experiments.

### 3.3. Characterization

UV-vis absorption and fluorescence spectroscopic measurements were taken by a Shimadzu UV-2600 UV-vis spectrophotometer and a Horiba FluoroMax-4 spectrofluorometer, respectively. The measurements were carried out by placing the AzPy solutions into a cleaned 1 cm quartz cell, followed by a fulling purge of the Nitrogen gas and sealing with parafilm^®^. Supercure-204S, Tokina, was used with a combination of Toshiba color filters, UV-35 + UV-D36A for UV light production (365 nm) and Y-43 + V-44 for a visible light generation (436 nm) to perform *trans*-to-*cis* and *cis*-to-*trans* photoisomerization experiments in monomer (~1.0 mW/cm^2^) and mixed turbid solution (~2.5 mW/cm^2^). Optical microscopy (OM) and fluorescence optical microscopy (FOM, λ_ex_ = 520–550 nm) images were recorded using an Olympus BX53 microscope. The height profile of the resulting microplates was plotted after taking a digital microscopy image with an oblique observation mode (OBQ) on DSX1000 digital microscope. Polarized optical microscopy (POM) investigation was performed using an Olympus BX53-P polarizing microscope (Olympus Corporation, Tokyo, Japan). Field-emission scanning electron microscopy (FE-SEM) images were captured with a TESKAN-MIRA3-LM microscope after platinum layer coating in a Hitachi E-1030 ion sputter for 60 s. The samples for microscopic observation were prepared by placing a small drop of AzPy mixed solutions on the cleaned glass substrate and used after fully drying the solvent at ambient temperature. X-ray diffraction (XRD) data were measured with CuKα radiation on Rigaku R-AXIS-IV and R-AXIS-VII X-ray imaging plate detectors.

## 4. Conclusions

In conclusion, we have developed pyrene-based self-assembled systems that present (i) stable emission intensity regardless of aggregation and (ii) concentration dependence of emission color from blue to yellow-orange. Our strategy for obtaining fluorescent sheet-like microstructures of varying size and completion degree began with the design of a pyrene-based chromophore (AzPy) in which sterically crowded azobenzene was introduced into pyrene. Excimer emission is clearly observed, along with monomer emission, in dilute polar solvents. The chromophore has a strong tendency to grow into sheet-like aggregates, from incomplete flakes to well-completed rectangular structures, depending on the concentration. The rectangular aggregates exhibit concentration dependence of emission wavelength from blue to yellow-orange. Our absorption and fluorescence spectroscopic results and OM, FOM, SEM, POM, and XRD observations reveal that inclined π–π stacking interactions (with relatively longer interplanar stacking distance) and high crystallinity, made possible with the aid of sterically twised azobenzene moiety, contributed to the expression of the interesting emission characteristics of the sheet-like microstructures. Our findings have important implications for the development of photoluminescent organic materials with desirable emission characteristics.

## Data Availability

The data are included within the manuscript and Supplementary Materials.

## Supplementary Materials

The following are available online at https://www.mdpi.com/article/10.3390/ijms24054504/s1.

## Sample Availability

Samples of AzPy are available from the corresponding author.
