# Supplementary material for: Role of Sterically Bulky Azobenzenes in the Molecular Assembly of Pyrene Derivatives: Rectangular Sheet-like Structures and Their Emission Characteristics
Source: Int J Mol Sci. 2023 Feb 24;24(5):4504. doi: 10.3390/ijms24054504 (PMC10003733; doi:10.3390/ijms24054504)
Supplement: Supplementary file 1 [file ijms-24-04504-s001.zip › ijms-2150072-supplementary.pdf]

Supplementary Materials (SM)

Role of Sterically Bulky Azobenzenes in the Molecular Assembly of Pyrene Derivatives: Rectangular Sheet-like Structures and Their Emission Characteristics

Pyae Thu and Mina Han

Contents

Figure S1. Changes in absorbances at 365 and 450 nm upon alternating irradiation with UV and blue light.

Figure S2. (a) Absorption spectra of **AzPy** in 50 μM DMF and DMF-H_2O. (b) Fluorescence spectra in 20 μM DMF and DMF-H_2O. (c) Plot of the length distribution of sheet-like structures obtained from 50 μM DMF-H_2O mixed turbid solution. (d) SEM image of incomplete thin structures.

Figure S3. OM image of incomplete rectangular structures obtained from a 20 μM DMF-H_2O mixed suspension.

Figure S4. (a) Absorption spectra of **AzPy** in 100 μM DMF and DMF-H_2O (1/1, v/v). (b) Fluorescence spectra of 100 and 920 μM (in DMF and DMF-H_2O).

Figure S5. Absorption spectra of **PyOH** in DMF (20 μM) and DMF-H_2O (1/4, v/v, 40 μM).

Figure S6. Absorption spectral changes of **AzPy** in DMF-H_2O (1/1, v/v, 50 μM) as a function of exposure time of UV light.

Figure S7. ^{1}H NMR spectrum of **AzPy**.

Figure S8. ^{13}C NMR spectrum of **AzPy**.

Figure S9. Synthesis of **PyBr** and its NMR data.

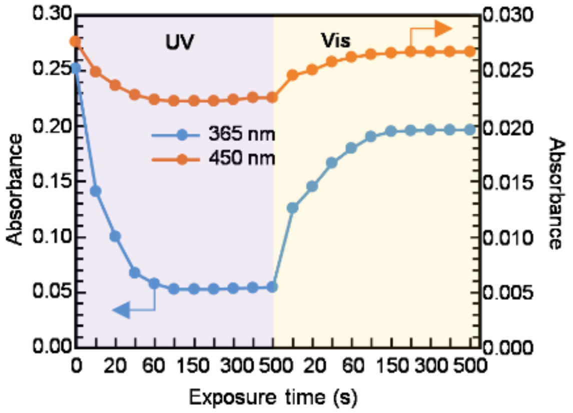

Figure S1. Changes in absorbances at 365 and 450 nm upon alternating irradiation with UV and blue light.

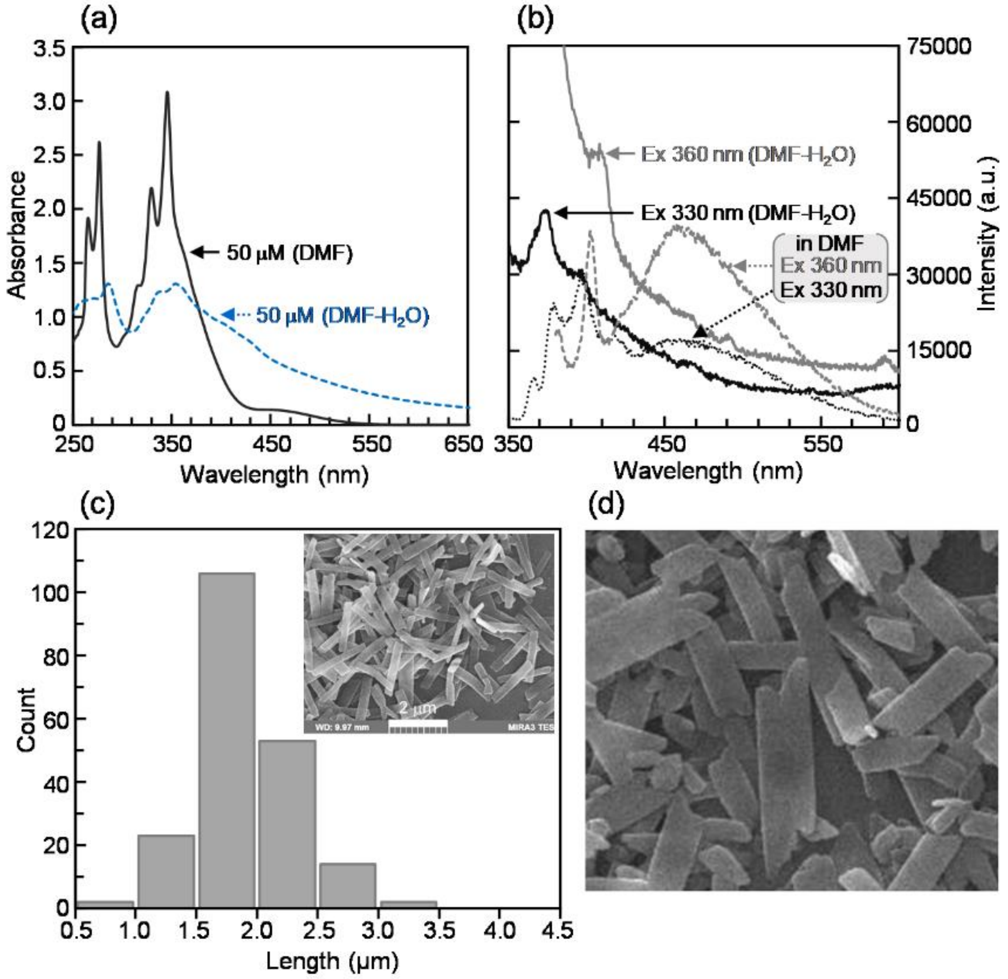

Figure S2. (a) Absorption spectra of **AzPy** in 50 μM DMF and DMF-H_2O (1/1, v/v). (b) Fluorescence spectra in 20 μM DMF and DMF-H_2O (1/3, v/v). (c) Plot of the length distribution of sheet-like structures obtained from 50 μM DMF-H_2O mixed turbid solution (counting 200 aggregates). (d) SEM image of incomplete thin structures. These aggregates seem to be formed in the early stage of self-assembly.

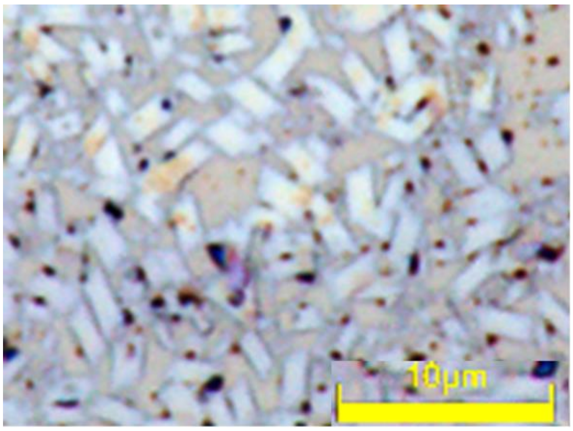

Figure S3. OM image of incomplete rectangular structures obtained from a 20 μM DMF-H_2O mixed suspension.

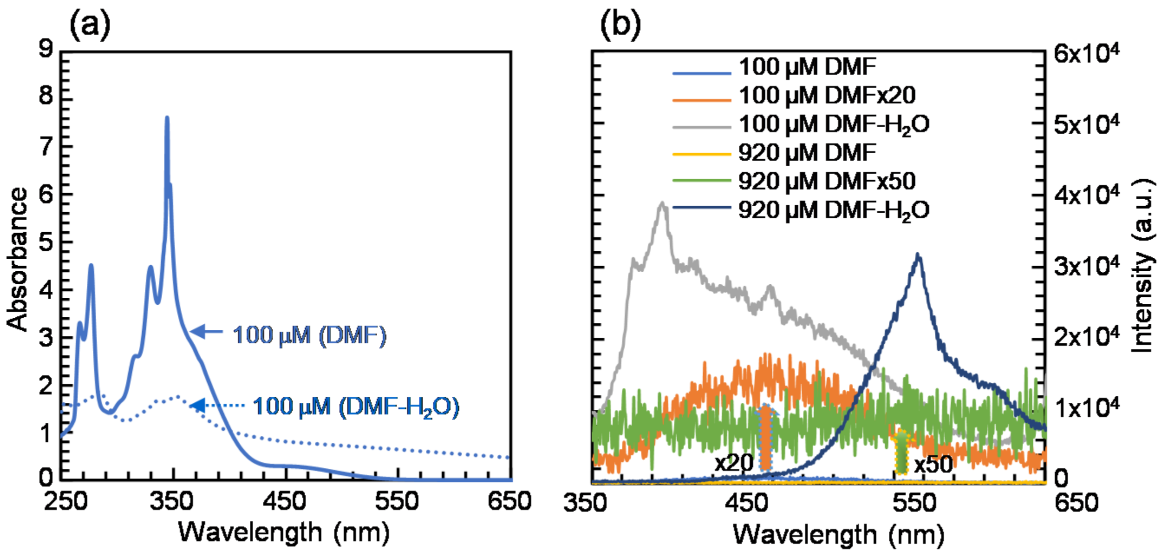

Figure S4. (a) Absorption spectra of **AzPy** in 100 μM DMF and DMF-H_2O (1/1, v/v). (b) Fluorescence spectra of 100 and 920 μM (in DMF and DMF-H_2O).

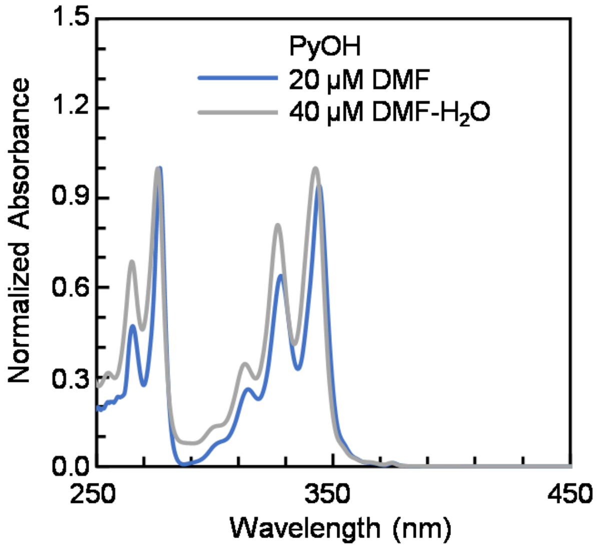

Figure S5. Absorption spectra of **PyOH** in DMF (20 µM) and DMF-H_2O (1/4, v/v, 40 µM).

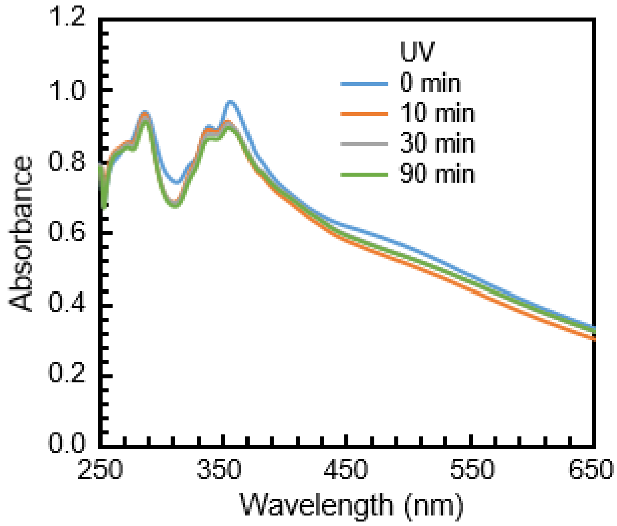

Figure S6. Absorption spectral changes of **AzPy** in DMF-H_2O (1/1, v/v, 50 µM) as a function of exposure time of UV light.

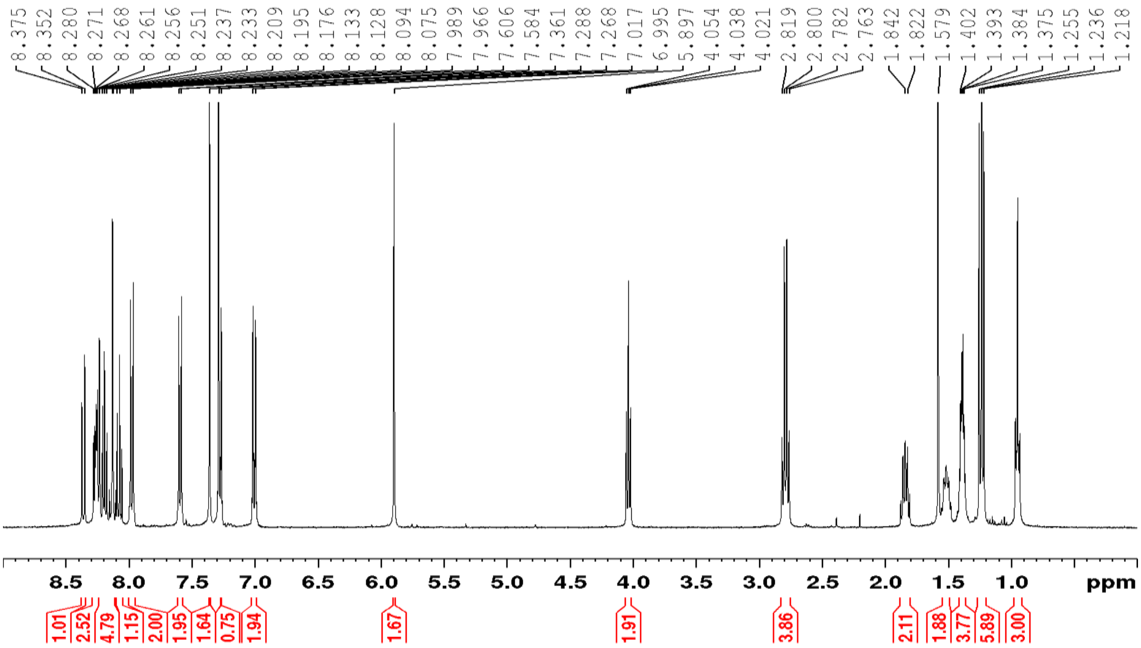

Figure S7. ^{1}H NMR spectrum of **AzPy** (in $CDCl_3$).

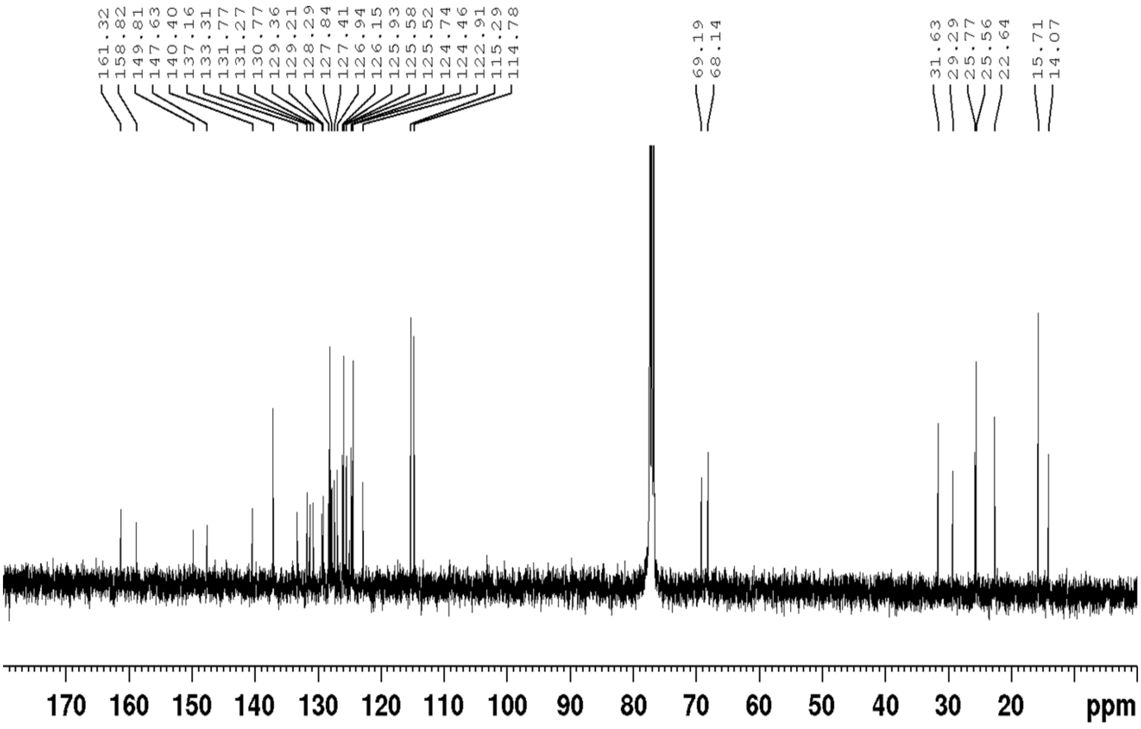

Figure S8. ^{13}C NMR spectrum of **AzPy** (in $CDCl_3$).

Synthesis of 1-Bromomethylpyrene **(PyBr)** [1]

Phosphorus tribromide (0.87 g, 3.23 mmol) was added to a suspension of commercial 1-pyrenemethanol (0.50 g, 2.15 mmol) in 5 mL of dry THF and stirred at room temperature for 2 h. The resulting mixture was washed with Et_2O and dried. The yellow solid (0.26 g, yield: 41 %) was obtained. ^{1}H NMR (400 MHz, $CDCl_3$) δ 8.31 (d, 1H, Py*H*), 8.15 (m, 3H, Py*H*), 7.98 (m, 5H, Py*H*), 5.18 (s, 2H, PyC*H*$_2$).

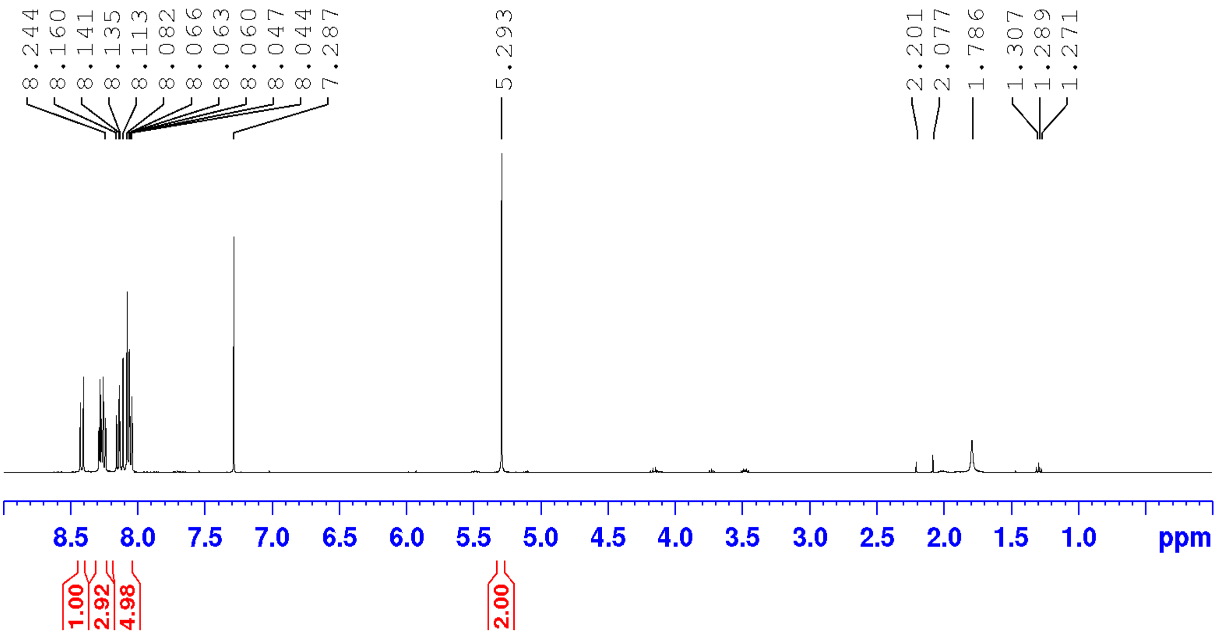

Figure S9. ^{1}H NMR spectrum of **PyBr** (in $CDCl_3$).

Reference

1. Park, S.Y.; Yoon, J.H.; Hong, C.S.; Souane, R.; Kim, J.S.; Matthews, S.E.; Vicens, J. A Pyrenyl-Appended Triazole-Based Calix [4] Arene as a Fluorescent Sensor for Cd^{2+} and Zn^{2+}. *J. Org. Chem.* **2008**, *73*, 8212-8218.
